# Variability Among Breast Cancer Risk Classification Models When Applied at the Level of the Individual Woman

**DOI:** 10.1007/s11606-023-08043-4

**Published:** 2023-02-07

**Authors:** Jeremy S. Paige, Christoph I. Lee, Pin-Chieh Wang, William Hsu, Adam R. Brentnall, Anne C. Hoyt, Arash Naeim, Joann G. Elmore

**Affiliations:** 1 Department of Radiology, University of California, Los Angeles, CA USA; 2 Department of Radiology, University of Washington School of Medicine, Seattle, WA USA; 3 Department of Medicine, Division of General Internal Medicine and Health Services Research, David Geffen School of Medicine, and Office of Health Informatics and Analytics, University of California, Los Angeles, Los Angeles, USA; 4 Centre for Evaluation and Methods, Wolfson Institute of Population Health, Charterhouse Square, Queen Mary University of London, London, UK; 5 Division of Hematology and Oncology, Department of Medicine, David Geffen School of Medicine, University of California, Los Angeles, CA USA; 6 Department of Medicine, Division of General Internal Medicine and Health Services Research and the National Clinician Scholars Program, David Geffen School of Medicine, University of California, Los Angeles, 1100 Glendon Ave, Ste. 900, Los Angeles, CA 90024 USA

**Keywords:** breast cancer, risk models, screening, mammography, chemoprevention

## Abstract

**Background:**

Breast cancer risk models guide screening and chemoprevention decisions, but the extent and effect of variability among models, particularly at the individual level, is uncertain.

**Objective:**

To quantify the accuracy and disagreement between commonly used risk models in categorizing individual women as average vs. high risk for developing invasive breast cancer.

**Design:**

Comparison of three risk prediction models: Breast Cancer Risk Assessment Tool (BCRAT), Breast Cancer Surveillance Consortium (BCSC) model, and International Breast Intervention Study (IBIS) model.

**Subjects:**

Women 40 to 74 years of age presenting for screening mammography at a multisite health system between 2011 and 2015, with 5-year follow-up for cancer outcome.

**Main Measures:**

Comparison of model discrimination and calibration at the population level and inter-model agreement for 5-year breast cancer risk at the individual level using two cutoffs (≥ 1.67% and ≥ 3.0%).

**Key Results:**

A total of 31,115 women were included. When using the ≥ 1.67% threshold, more than 21% of women were classified as high risk for developing breast cancer in the next 5 years by one model, but average risk by another model. When using the ≥ 3.0% threshold, more than 5% of women had disagreements in risk severity between models. Almost half of the women (46.6%) were classified as high risk by at least one of the three models (e.g., if all three models were applied) for the threshold of ≥ 1.67%, and 11.1% were classified as high risk for ≥ 3.0%. All three models had similar accuracy at the population level.

**Conclusions:**

Breast cancer risk estimates for individual women vary substantially, depending on which risk assessment model is used. The choice of cutoff used to define high risk can lead to adverse effects for screening, preventive care, and quality of life for misidentified individuals. Clinicians need to be aware of the high false-positive and false-negative rates and variation between models when talking with patients.

**Supplementary Information:**

The online version contains supplementary material available at 10.1007/s11606-023-08043-4.

## INTRODUCTION

Breast cancer risk models estimate a woman’s risk of developing invasive breast cancer over a defined period, most commonly 5 years. These models have been developed and validated at a population level,^1^ and their use for individual patients in clinical practice is encouraged. These risk models differ in the number and weighting of variables included in their calculations. While age is the strongest predictor in all models, other components are variable and may include family history, genetic carrier status, and nongenetic risk factors such as breast density and previous breast biopsy.^2^ These commonly used risk prediction models have similar accuracy and good discrimination when assessed in large populations.^3–5^

While current breast cancer risk assessment tools work well at a population level, little attention has been paid to how they perform at an individual level or to the impact of different thresholds for defining high risk. As we move toward a precision medicine approach in healthcare, these risk models are increasingly being used to identify women who would benefit from chemoprevention^6–11^ and supplemental MRI screening.^12–15^ In 2019, the US Preventive Services Task Force recommended that clinicians offer risk-reducing medications, such as tamoxifen, raloxifene, or aromatase inhibitors, to women who are at high risk for breast cancer in the next 5 years and at low risk for adverse medication effects.^10^ While prior recommendations and clinical trials have supported using a 5-year risk cutoff of 1.67%, the Task Force in 2019 recommended a new cutoff of 3%. Previous publications have not adequately assessed the variation in risk estimates for the ≥ 3.0% 5-year threshold at the level of the individual. The few studies evaluating differences in model performance at the level of the individual woman have used small sample sizes and only evaluate lifetime risk,^16,17^ or report results for a 5-year threshold of ≥ 1.67%.^18^ Since key screening and medical intervention decisions are based on a woman’s estimated risk of developing invasive breast cancer, a consistent and accurate risk estimate is essential.

The objective of this study was to evaluate the accuracy and extent of disagreement of three common clinical risk models at the individual level when using these two different cutoffs (≥ 1.67% and ≥ 3.0%) to define high risk of breast cancer at 5 years. We focused on the models most likely to be used across all clinical specialties including, importantly, primary care and general internal medicine providers. The three risk models evaluated in this paper are the Breast Cancer Risk Assessment Tool (BCRAT, also called the Gail model),^19^ the Breast Cancer Surveillance Consortium (BCSC),^20,21^ and the International Breast Intervention Study (IBIS, also called the Tyrer-Cuzick model).^22^ The BCRAT and IBIS are thought to be the two most commonly used models in clinical practice in the USA.^23,24^ Moreover, these three models all have readily accessible simple online calculators and are recommended for use in primary care.^25^ In addition to assessing inter-model disagreement at the level of the individual, we used a subset of women with long-term cancer outcomes from cancer registry linkage to assess the accuracy of the risk models, as well as model calibration and discrimination at the population level.

## METHODS

Data from UCLA Health, a diverse, multisite US health system serving a large geographic region of Southern California, were used for this study. Data were collected as part of the Athena Breast Health Network, a statewide quality improvement initiative across the University of California medical and cancer centers.^26^ Women participated at the time of mammography screening by completing informed consent and digital surveys that assessed their health history, lifestyle behaviors, and family history of cancer (IRB #10-001083). If a woman had multiple mammography examinations during the study period and thus filled out multiple surveys, the earliest most complete survey with the least amount of missing information was used. Overall, 50 variables were extracted from the survey and electronic health record to generate a dataset to use for the three risk models in this study. The key variables are listed in Table 1 and Supplementary Table 1. As data on body mass index (BMI) and mammographic density were not included in the original self-reported survey data, we acquired these data from participants’ electronic health records. We calculated BMI by using height and weight recorded within 1 year of the survey date. Mammographic density (BI-RADS^TM^) was acquired from the mammography report. If multiple mammography screening exams were available for a woman, we used the one closest to the date when the woman completed the survey.

**Table 1:** Patient Characteristics and Risk Factors for Women Presenting for Screening Mammography Between 2011 and 2015 (*N* = 31,115)

Characteristic	All patients (*N* = 31,115)
Age, mean ± SD (range)*	55.3 ± 9.6 (40–74)
Age at menarche, mean ± SD (range)*	12.7 ± 1.5 (9–17)
Age at first live birth, mean ± SD (range)*	31.9 ± 7.3 (19–41)
Age groups	
40–49	10,350 (33.3%)
50–59	9699 (31.2%)
60–69	8335 (26.8%)
70–74	2731 (8.8%)
Race or ethnicity	
Non-Hispanic White	16,776 (53.9%)
Non-Hispanic Black	2545 (8.2%)
Hispanic	3053 (9.8%)
Asian	3418 (11.0%)
Other	2718 (8.7%)
Unknown	2605 (8.4%)
Age at menarche	
11 and younger	5844 (18.8%)
12–13	17,168 (55.2%)
More than 13	8103 (26.0%)
Body mass index (kg/m^2^)	
18–24	14,007 (45.0%)
25–29	6552 (21.1%)
30 and above	5066 (16.3%)
Unknown	5490 (17.6%)
Age at first live birth	
< 20	1914 (6.2%)
20–24	3334 (10.7%)
25–29	5667 (18.2%)
30 and above	9087 (29.2%)
Nulliparous	10,754 (34.6%)
Unknown	359 (1.2%)
Menopausal status	
Pre-menopausal	10,950 (35.2%)
Peri-menopausal	1268 (4.1%)
Post-menopausal	18,110 (58.2%)
Unknown	787 (2.5%)
Menopausal hormone therapy use	
Never or unknown	29,412 (94.5%)
Ever	1703 (5.5%)
Personal history of benign breast disease	
No	3278 (10.5%)
Yes	235 (0.8%)
Unknown	27,602 (88.7%)
Mammographic density	
Almost entirely fatty	4445 (14.3%)
Scattered fibroglandular	16,181 (52.0%)
Heterogeneously dense	8397 (27.0%)
Extremely dense	2092 (6.7%)
Result of biopsy	
No prior biopsy	27,335 (87.9%)
Prior biopsy but diagnosis unknown	3545 (11.4%)
Atypical hyperplasia	235 (0.8%)
First-degree relatives with breast cancer	
0/NA	26,158 (84.1%)
1	4619 (14.8%)
2 and above	338 (1.1%)
Second-degree relatives with breast cancer	
0/NA	23,995 (77.1%)
1	5923 (19.0%)
2 and above	1197 (3.8%)

Unless otherwise indicated, data are number of patients with percentages in parentheses. Age is recorded in years

*NA* not applicable, *SD* standard deviation

*Data are means ± standard deviation, with range in parentheses

All women aged 40 to 74 years presenting for screening mammography between January 1, 2011, and December 31, 2015, were considered for inclusion for the primary analysis. For a subgroup of women who entered the study from 2011 to 2013, a minimum of five full years of follow-up data were available and breast cancer outcome data for these women were collected from the health system and regional cancer registry linkage. For women with invasive breast cancer, the last follow-up date is the date of diagnosis. For non-cancer cases, a full 5 years of follow-up data were available from the date of their survey. We identified 382 cases of invasive cancer in these asymptomatic women presenting for screening mammograms, of which 16 women died within 5 years, making the 5-year survival 97%. This 5-year survival is similar to that reported in other studies reporting the survival of women with invasive breast cancer detected by screening mammography.^27–29^

The study population included a mix of women on annual and biennial screening intervals, with more women screened in the health system in later years as capacity expanded. Patients were offered enrollment into the Athena Breast Health Network at each screening exam; therefore, patients may have only answered the survey questionnaire once but may still have had more than one subsequent screening exam during the study period. Women were excluded from our analysis if they had a previous diagnosis of invasive breast cancer or ductal carcinoma in situ (DCIS), prior breast augmentation, or prior mastectomy.

BCRAT software (Breast Cancer Risk Assessment SAS Macro, Version 4, Gail Model) was downloaded from the National Institute of Health, Division of Cancer Epidemiology & Genetics.^30^ BCSC software was downloaded from the Breast Cancer Surveillance Consortium.^31^ IBIS software (IBIS Breast Cancer Risk Evaluation Tool, version 8) was downloaded from the Wolfson Institute of Preventative Medicine.^32^ Missing data for the IBIS model were prepared according to the instructions provided on the IBIS website (https://ems-trials.org/riskevaluator/).

### Statistical Analysis

Five-year breast cancer risk was calculated using the three different models for each woman. Women were divided into average- or high-risk groups for 5-year risk estimates using the cutoffs of ≥ 1.67% and ≥ 3.0%, the two values used by clinicians when considering chemoprevention treatment recommendations.^10^ The “average-risk” group includes what are likely to be both low- and average-risk women, two terms that are not well defined for 5-year risk by the existing models. Therefore, for the purpose of this study, we have grouped any women who were not high risk under the term “average risk.”

We describe self-reported demographics and key breast cancer risk factors obtained at the time of imaging. Two-sided hypothesis testing on descriptive statistics employed chi-squared testing for categorical variables and non-parametric Wilcoxon test for continuous variables. We calculated population-level model performance based on calibration and discrimination for the subset of women with full cancer outcome data at 5 years. We assessed each model’s ability to discriminate between women who did and did not develop invasive breast cancer within 5 years by estimating and testing for differences in the C-statistic and plotting ROC curves. Calibration was assessed using both observed probability and predicted scores for 5-year breast cancer outcome from three models and plots by risk decile. Expected value is calculated as the sum of the predicted 5-year absolute risk estimated from each risk model. Observed value and the 95% confidence interval were calculated for a Poisson distribution. To compare binary 5-year risk between breast cancer risk models, we describe pairwise comparison in 2 × 2 tables. SAS version 9.4 (SAS Institute, NC) was used to perform all statistical analyses.

## RESULTS

Over the 5-year study period, 48,980 women presenting for mammography were surveyed, of which 36,438 women met the eligibility criteria for risk models, with 85.4% (31,115) of eligible women having the minimum recorded risk data to run all three models (age, age at menarche, breast density, and history of breast biopsy [Supplementary Figure 1]). The clinical variables included in each risk model and percentage with complete data for each risk factor are shown in Supplementary Table 1. Overall, 26,170 women (84.1%) had complete data on the 7 BCRAT risk factors, 26,391 (84.8%) had complete data on the 5 BCSC risk factors, and 26,170 (84.1%) had complete risk factor data for both BCRAT and BCSC models. No woman in our cohort had complete data for the IBIS model, which uses 84 questions about immediate and non-immediate family members as inputs. Among the subgroup of women who had 5 years of follow-up (*N* = 11,589), 382 (3.3%) had an invasive breast cancer diagnosis within 5 years.

### Patient Characteristics and Population-Level Breast Cancer Risk Assessment

Characteristics of the 31,115 women are shown in Table 1. For the overall population, the percentage of women identified as having ≥ 1.67% 5-year risk for developing invasive breast cancer was 35.5%, 20.0%, and 27.4% for BCRAT, BCSC, and IBIS, respectively, while the percent identified as having ≥ 3.0% 5-year risk was 6.6%, 2.6%, and 6.6%, respectively, for the same three models (Table 2). To test for the impact of missing data, a sensitivity analysis was conducted to examine the high-risk proportion for patients with complete data (*N* = 26,170) for both the BCRAT and BCSC models. The percentages of 5-year high risk from this complete data were similar to that from the full data (Supplementary Table 2).

**Table 2:** Five-Year Risk Estimates for Women Assessed Across Three Different Breast Cancer Risk Models (*N* = 31,115)

Risk model	Cutoff ≥ 1.67%		Cutoff ≥ 3.0%	
	Average risk (< 1.67%)	High risk (≥ 1.67%)	Average risk (< 3.0%)	High risk (≥ 3.0%)
BCRAT	20,069 (64.5%)	11,046 (35.5%)	29,067 (93.4%)	2048 (6.6%)
BCSC	24,903 (80.0%)	6212 (20.0%)	30,295 (97.4%)	820 (2.6%)
IBIS	22,604 (72.6%)	8511 (27.4%)	29,052 (93.4%)	2063 (6.6%)

Data are number of patients with percentages in parentheses

*BCRAT* Breast Cancer Risk Assessment Tool, *BCSC* Breast Cancer Surveillance Consortium, *IBIS* International Breast Cancer Intervention Study model

### Discordance Between the Models at the Level of the Individual

**Figure 1:**
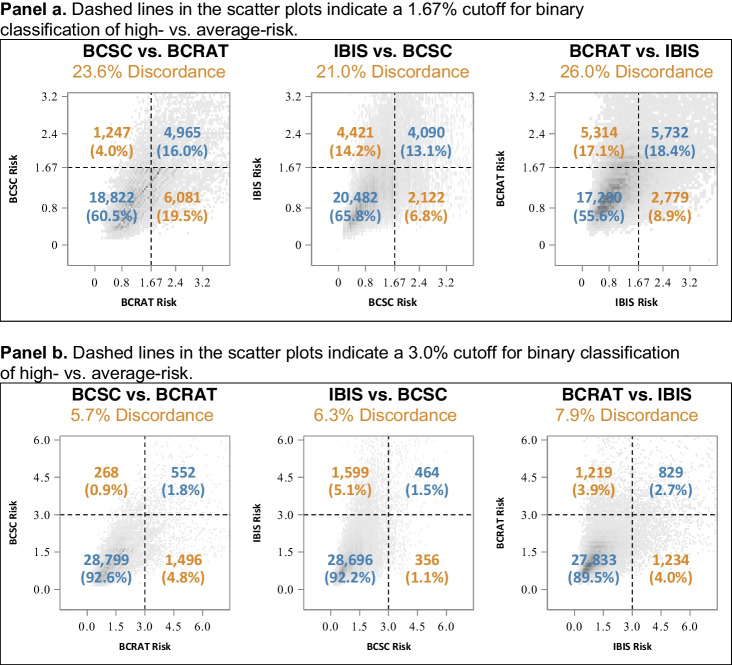
Pairwise comparisons of an individual woman’s risk of being told she is at “high risk” of a breast cancer diagnosis within 5 years when using the three commonly used risk models (*N* = 31,115). * **Panel a.** Dashed lines in the scatter plots indicate a 1.67% cutoff for binary classification of high- vs. average-risk. **Panel b.** Dashed lines in the scatter plots indicate a 3.0% cutoff for binary classification of high- vs. average-risk. *The sum of the percentages may not add up to 100% due to rounding**.**

Pairwise comparisons of the individual women’s 5-year risk estimates across the three risk models are shown in Figure 1. When the risk cutoff was ≥ 1.67%, and two models were used, more than one in five women received a different risk classification (discordance values ranging from 21.0 to 26.0%). For example, when comparing the risk estimates from BCSC versus BCRAT models, 23.6% discordance was noted (i.e., almost one in four women were labeled “high risk” by one model but not the other). IBIS versus BCRAT models had 26.0% discordance. IBIS versus BCSC models had 21.0% discordance. When using the cutoff of ≥ 3.0% and two models were used, more than 5% of women received a different risk classification (with discordance values ranging from 5.7 to 7.9%).

**Figure 2:**
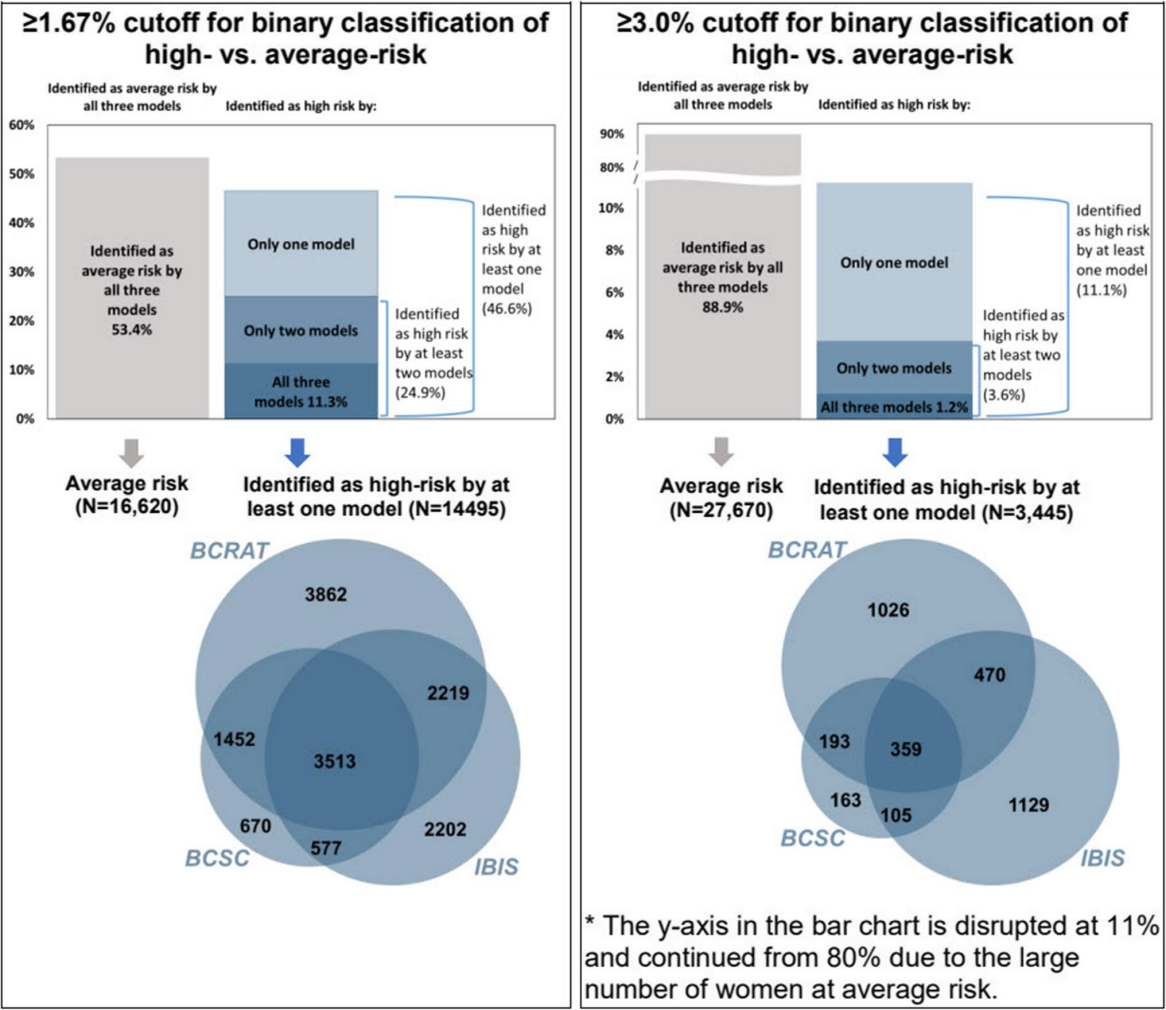
Agreement of breast cancer risk model results for individual women identified as high risk within 5 years on at least one of the breast cancer risk prediction models (*N* = 31,115). The Venn diagram at the bottom presents data for all women identified as high risk by at least one model, using shaded overlapping regions to represent the Boolean operation. The size of each Venn diagram circle corresponds to the number of women identified as high risk by each risk model**.**

Agreement of the risk model results for individual women identified as high risk within 5 years on one, two, or all three of the models is shown in Figure 2. When evaluating the individual’s risk of being categorized as high risk using all three models, and using a cutoff of ≥ 1.67%, 46.6% (14,495) of women were identified as having high 5-year risk by at least one of the models, 24.9% (7761) were identified as having high 5-year risk on at least two of the models, and 11.3% (3513) were identified as having high 5-year risk by all three models. The proportion of women noted as high risk for the cutoff point of ≥ 3.0% was lower, with 11.1% (3445) of women identified as having high 5-year risk by at least one of the models, 3.6% (1127) by at least two models, and 1.2% (359) by all three models.

### Accuracy of Risk Classification in the Subgroup of Women with 5 Years of Follow-Up

**Figure 3:**
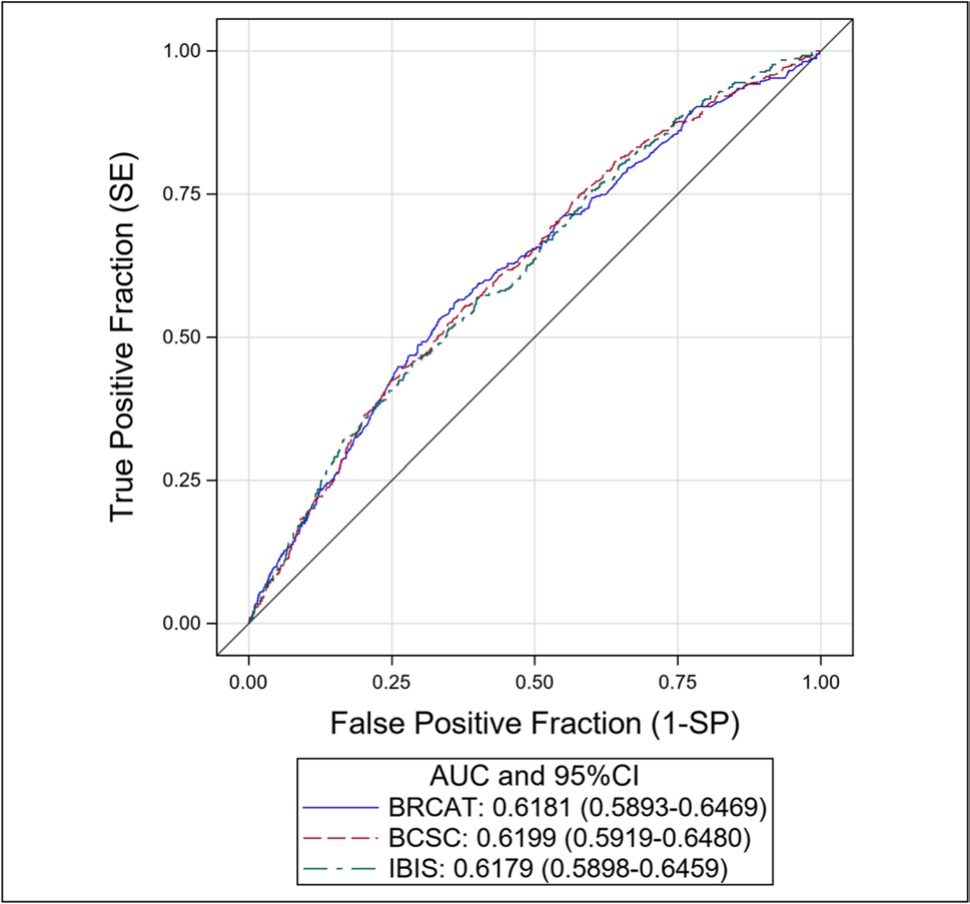
Receiver operating characteristic curve (ROC curve) illustrating the similar population-level performance of the three models for 5-year breast cancer risk prediction (*N* = 11,589 with 5 years of follow-up data)**.**

All three models performed similarly at a population level with similar discriminatory power and calibration plots in the subgroup of women with 5 years of follow-up data (Fig. 3 and Supplementary Figure 2). Supplementary Table 3 summarizes baseline characteristics between women with and without subsequent breast cancer diagnosis.

The accuracy of classifications when using these risk models is shown in Table 3. When using the ≥ 1.67% threshold, the sensitivity of the models ranged from 37.7 to 56.3% and specificity ranged from 63.7 to 77.9%. As an example, when the BCRAT was applied to the 11,589 women, about half of the women diagnosed with breast cancer within 5 years were correctly identified as high risk using the ≥1.67% threshold (215/382; true-positive rate: 56.3%) while over 4000 women who did not develop invasive breast cancer were classified as high risk (false-positive rate: 36.3%). When using the more conservative ≥ 3.0% cutoff, the sensitivity of the three models dropped to a range of 6.3 to 17.0% and specificity increased to a range of 91.3 to 96.7%. When the BCRAT was used, a much smaller number of women with breast cancer were identified as high risk (49/382; 12.8%), thus misclassifying most of the women with breast cancer, yet there was also a much smaller number of women who did not develop invasive breast cancer incorrectly classified as high risk using this cut-point (*N* = 740; 6.6%).

**Table 3:** Accuracy of 5-Year Breast Cancer Risk Prediction by Model in the Subgroup of Women with 5 Years of Follow-Up Data (*N* = 11,580)

Risk model	Sensitivity	Specificity	Positive predictive value	Negative predictive value
≥ 1.67% cutoff for binary classification of 5-year high vs. average risk				
BCRAT	56.3 (51.3–61.3)	63.7 (62.8–64.6)	5.0 (4.4–5.7)	97.7 (97.4–98.1)
BCSC	37.7 (32.8–42.6)	77.9 (77.1–78.6)	5.5 (4.6–6.4)	97.3 (97.0–97.7)
IBIS	46.9 (41.9–51.9)	68.8 (68.0–69.7)	4.9 (4.2–5.6)	97.4 (97.1–97.8)
≥ 3.0% cutoff for binary classification of 5-year high vs. average risk				
BCRAT	12.8 (9.5–16.2)	93.4 (92.9–93.9)	6.2 (4.5–7.9)	96.9 (96.6–97.2)
BCSC	6.3 (3.8–8.7)	96.7 (96.4–97.1)	6.2 (3.8–8.5)	96.8 (96.5–97.1)
IBIS	17.0 (13.2–20.8)	91.3 (90.8–91.8)	6.2 (4.8–7.7)	97.0 (96.7–97.3)

Data are percentages with 95% confidence intervals in parentheses

*BBCRAT* Breast Cancer Risk Assessment Tool, *BCSC* Breast Cancer Surveillance Consortium, *IBIS* International Breast Cancer Intervention Study model

## DISCUSSION

Breast cancer risk models are increasingly used in clinical care to provide more personalized, risk-based screening and prevention recommendations. In our analysis, we found substantial variability in categorizing individuals as high risk for developing invasive breast cancer based on the risk model and the 5-year cutoff used. Thus, women are likely receiving vastly different recommendations depending on which breast cancer risk model is used and which cutoff is used to define “high risk.” While these models perform similarly at the population level, as noted in our study and in prior studies,^3–5,18,33,34^ our analyses highlight marked disagreement between models in who they identify as “high risk.” For example, more than 20% of women would be classified as high risk of developing invasive breast cancer in the next 5 years by one model but average risk by a second model if using the ≥ 1.67% threshold. We found that if all three models were used, almost half of women would be considered at high 5-year risk by at least one model when using the ≥ 1.67% cutoff. However, most women will not be diagnosed with breast cancer within 5 years, thus leading to many women being incorrectly classified as high risk.

These findings highlight the tradeoff of sensitivity and inaccurate classification as being “high risk” when using the two different thresholds currently recommended to define high risk. For example, when using the ≥ 1.67% cutoff for considering chemoprevention, about half of the women diagnosed with a future breast cancer might be correctly identified as high risk, yet many more women will be falsely classified as high risk. When using the more conservative ≥ 3.0% cutoff, there will be a much smaller number of women incorrectly classified as high risk, yet most of the women with a future breast cancer diagnosis will be missed.

We focused on the BCRAT, BCSC, and IBIS models as they are commonly used models, readily accessible online, and recommended for use in primary care.^23–25^ In contrast, more specialized models such as BOADICEA (CanRisk) and BRCAPRO, while well validated, are recommended for use by genetics specialists or designed to assess the probability of specific genetic risk factors.^35–38^ Whether or not our findings of variable high-risk categorization at the individual level holds across these additional models should be explored in future studies.

While the current practice of shared decision-making based on women’s breast cancer risk estimates from these commonly used risk models is not precise, newer risk models are being developed. Some models are now including information on breast cancer susceptibility genes and genetic susceptibility variants, while several recent studies suggest that quantitative imaging biomarkers and artificial intelligence algorithms might also supplement or supplant the current, subjective clinical risk assessment tools.^39–42^ Risk prediction based on objective imaging-based deep learning breast cancer risk models has the potential to remove the subjectivity involved with the use of self-reported risk information that currently drives risk-based screening and prevention practices.^43,44^ However, these new tools require having had a screening mammography examination and thus may not be useful to assess risk in the context of deciding what age to start screening.^45^

This study has several limitations. The cohort was drawn from women enrolled in a longitudinal screening study. Although we had extensive risk factor data on many participants, some family history was missing as was data on polygenetic risk scores. Despite this, the percentage of women identified as high risk was similar for both those with complete and incomplete (minimum required) data for the BCRAT and BCSC models, suggesting that the most impactful risk factors were recorded for most women. Moreover, we suspect that the results based on the minimum number of risk factors required to calculate each model are more representative of how these models are likely used in real-world clinical practice, where risk factor data are typically self-reported. Several prior studies have demonstrated inconsistencies in a woman’s accuracy at remembering age of menstruation and other historical data.^46,47^ Finally, BCRAT and BCSC were developed to evaluate risk of invasive breast cancer, yet IBIS was developed to evaluate risk of invasive or ductal carcinoma in situ. In order to compare across models, we used the outcome of invasive breast cancer only for all three models.^48,49^

Our study has multiple strengths. First, we considered the differences in model performance at the individual level. The few studies that have done this are limited by small sample size, did not evaluate disagreement between models when a woman uses more than one risk model, or did not provide comparative evaluation using the more conservative 5-year risk threshold of ≥ 3.0%. Our analysis also uses data from a large racially and ethnically diverse population of women.

In summary, we noted substantial discordance in how three commonly used clinical risk models predicted 5-year invasive breast cancer risk, especially when using the cutoff of ≥ 1.67% to define high risk. While risk models are generally considered useful in determining eligibility for high-risk screening and intervention at the population level, they may be less useful for guiding discussions about screening and interventions at the individual level. Our findings highlight the risk of a blanket approach to using risk prediction models to inform individual-level medical screening and treatment decisions. Future research efforts are necessary to improve on the ability of these risk models to inform shared decision-making for individual patients.

## Supplementary Information

ESM 1 (DOCX 105 kb)

## Data Availability

The data underlying this article cannot be shared publicly due to privacy and ethical considerations for the individuals that participated in the study. The data will be shared on reasonable request to the corresponding author.

## Declarations

### Disclaimer

Pin-Chieh Wang, University of California, Los Angeles, conducted the data analysis. P. Wang had full access to all the data in the study and takes responsibility for the integrity of the data and the accuracy of the data analysis.
