# Supplementary material for: Variability Among Breast Cancer Risk Classification Models When Applied at the Level of the Individual Woman
Source: J Gen Intern Med. 2023 Feb 7;38(11):2584–92. doi: 10.1007/s11606-023-08043-4 (PMC10465429; doi:10.1007/s11606-023-08043-4)
Supplement: Supplementary file 1 — (DOCX 105 kb) [file 11606_2023_8043_MOESM1_ESM.docx]

**ELECTRONIC APPENDIX**

**Paper Title:** Variability Among Breast Cancer Risk Classification Models When Applied at the Level of the Individual Woman

**Author:** Jeremy S. Paige, MD, PhD, et al

**TABLE OF CONTENTS**

Supplementary Table 1. Summary of inputs used to calculate the different breast cancer risk models and percentage available data for each input. (n=31,115) 3

Supplementary Table 2. Sensitivity analysis for patients categorized as high-risk for breast cancer with complete data for 8 risk factors for both BCRAT and BCSC models 5

Supplementary Table 3. Patient characteristics and risk factors by breast cancer diagnosed within 5 years for patients presenting for screening mammography between 2011 and 2013 (n=11,589 with 5 years of follow-up data) 6

Supplementary Figure 1. Flow chart of patient selection criteria 8

Supplementary Figure 2. Calibration plots by decile of risk for 5-year predictions 9

**Supplementary Table 1. Summary of inputs used to calculate the different breast cancer risk models and percentage available data for each input. (N=31,115).**

|  | **BCRAT Model**  **(Gail)** | **BCSC Model** | **IBIS Model**  **(Tyrer-Cuzick)** ‡ |
| --- | --- | --- | --- |
| **Personal information** | | | |
| Current age* | Yes^†^ (100%) | Yes^†^ (100%) | Yes^†^ (100%) |
| Previous breast biopsy* | Yes (100%) | Yes^†^ (100%) | Yes (100%) |
| History of atypical hyperplasia | Yes (99%) | Yes (99%) | Yes (99%) |
| Race or ethnicity | Yes (92%) | Yes (92%) | NA |
| Age at menarche* | Yes^†^ (100%) | NA | Yes (100%) |
| Age at first birth | Yes (99%) | NA | Yes (99%) |
| Parity | NA | NA | Yes (99%) |
| Menopausal status | NA | NA | Yes (98%) |
| Menopausal hormone therapy use | NA | NA | Yes (100%) |
| Body-mass index | NA | NA | Yes (82%) |
| Genetic test | NA | NA | Yes (83%) |
| History of lobular carcinoma in situ | NA | Yes (100%) | Yes (100%) |
| Mammographic density* | NA | Yes^†^ (100%) | Yes (100%) |
| **Information about the individual and their family members** | | | |
| First-degree relatives with breast cancer | Yes (85%) | Yes (85%) | Yes (85%) |
| Second-degree and third-degree relatives with breast cancer | NA | NA | Yes (80%) |
| Age at breast cancer diagnosis for first-degree relatives | NA | NA | Yes (72%) |
| Bilateral breast cancer for first-degree relatives | NA | NA | Yes (71%) |
| Ovarian cancer for first-degree relatives | NA | NA | Yes (65%) |
| Vital status of family members | NA | NA | Yes (72%) |
| BRCA1 and BRCA2 mutation status for family members | NA | NA | Yes (11%) |
| Ashkenazi Jewish heritage | NA | NA | Yes (100%) |
| Note—These models do not require all data inputs for calculation of a risk estimate.  ^*^ Minimum number of recorded data required to calculate risk from all three models used in this study, and therefore input data required for eligibility for this study.  ^†^ Inputs required to calculate risk from each individual risk model.  ‡ The detail of available data from survey and clinical data for IBIS model calculation includes Current age (of patient), Age at menarche, Number of live births, Age at first birth, Menopausal status, Age at menopause, Height, Weight, History of atypical hyperplasia, Ashkenazi Jewish heritage, HRT use , Current type of HRT taken , Length of time taking HRT in the past, Genetic testing of the woman , Has the mother had breast cancer?, Has the mother had ovarian cancer?, Age at which mother developed breast cancer, if not then their current age or the age at which they died, Number of sisters , Has the sister had breast cancer?, Has the sister had ovarian cancer?, Age at which sister developed breast cancer, or if not then their current age or the age at which they died, Has the paternal gran had breast cancer?, Has the paternal gran had ovarian cancer?, Has the maternal gran had breast cancer?, Has the maternal gran had ovarian cancer?, Number of paternal aunts, Has the paternal aunt had breast cancer?, Has the paternal aunt had ovarian cancer?, Number of maternal aunts, Has the maternal aunt had breast cancer?, Has the maternal aunt had ovarian cancer?, Number of daughters, Has the daughter had breast cancer?, Has the daughter had ovarian cancer?, Density type, BI-RADS density | | | |

**Supplementary Table 2. Sensitivity analysis for patients categorized as high-risk for breast cancer with complete data on required risk factors for both BCRAT and BCSC models.***

|  | **% Assessed as High Risk Using**  **5-Year Risk ≥1.67%** | | **% Assessed as High Risk**  **Using**  **5-Year Risk ≥3%** | |
| --- | --- | --- | --- | --- |
| **Risk Model** | **All Patients**  **(N = 31,115)** | **Patients with Complete Data**  **(N = 26,170)** | **All Patients**  **(N = 31,115)** | **Patients with Complete Data**  **(N = 26,170)** |
| BCRAT | 11046 (35.5%) | 9669 (37.0%) | 2048 (6.6%) | 1952 (7.5%) |
| BCSC | 6212 (20.0%) | 5011 (19.2%) | 820 (2.6%) | 699 (2.7%) |

^*^ Results are shown only for women with all required risk factors for both BCRAT and BCSC models. No woman in our cohort had complete data for the IBIS model, which uses 84 questions about immediate and non-immediate family members as inputs.

**Supplementary Table 3. Patient characteristics and risk factors by breast cancer diagnosed within 5 years for patients presenting for screening mammography between 2011 and 2013 (n=11,589 with 5 years of follow-up data).**

| ***Characteristic*** | | ***All Patients (N=11589)*** | ***No breast cancer (N=11207)*** | ***With breast Cancer^†^ (N=382)*** | ***P-value^‡^*** |
| --- | --- | --- | --- | --- | --- |
|  | |  |  |  |  |
| Age, Mean ± SD (Range)^*^ | | 55.3 ± 9.4 (40-74) | 55.2 ± 9.3 (40-74) | 58.3 ± 9.5 (40-74) | <.01^*^ |
| Age at menarche, Mean ± SD (Range)^*^ | | 12.8 ± 1.5 (9-17) | 12.8 ± 1.5 (9-17) | 12.7 ± 1.6 (9-17) | 0.14 |
| Age at first live birth, Mean ± SD (Range)^*^ | | 32.3 ± 7.0 (19-41) | 32.3 ± 7.0 (19-41) | 32.6 ± 6.7 (19-41) | 0.28 |
| Age Groups | |  |  |  | <.01 |
| 40-49 | | 3835 (33.1%) | 3745 (33.4%) | 90 (23.6%) |  |
| 50-59 | | 3776 (32.6%) | 3664 (32.7%) | 112 (29.3%) |  |
| 60-69 | | 2974 (25.7%) | 2859 (25.5%) | 115 (30.1%) |  |
| 70-74 | | 1004 (8.7%) | 939 (8.4%) | 65 (17.0%) |  |
| Race or ethnicity | |  |  |  | 0.14 |
| Non-Hispanic White | | 6641 (57.3%) | 6411 (57.2%) | 230 (60.2%) |  |
| Non-Hispanic Black | | 912 (7.9%) | 886 (7.9%) | 26 (6.8%) |  |
| Hispanic | | 1083 (9.3%) | 1056 (9.4%) | 27 (7.1%) |  |
| Asian | | 774 (6.7%) | 739 (6.6%) | 35 (9.2%) |  |
| Other | | 1360 (11.7%) | 1320 (11.8%) | 40 (10.5%) |  |
| Unknown | | 819 (7.1%) | 795 (7.1%) | 24 (6.3%) |  |
| Age at menarche | |  |  |  | 0.36 |
| 11 and younger | | 2104 (18.2%) | 2025 (18.1%) | 79 (20.7%) |  |
| 12-13 | | 6409 (55.3%) | 6209 (55.4%) | 200 (52.4%) |  |
| More than 13 | | 3076 (26.5%) | 2973 (26.5%) | 103 (27.0%) |  |
| Body mass index (kg/m^2^) | |  |  |  | 0.27 |
| 18-24 | | 5546 (47.9%) | 5364 (47.9%) | 182 (47.6%) |  |
| 25-29 | | 2377 (20.5%) | 2309 (20.6%) | 68 (17.8%) |  |
| 30 and above | | 1782 (15.4%) | 1715 (15.3%) | 67 (17.5%) |  |
| Unknown | | 1884 (16.3%) | 1819 (16.2%) | 65 (17.0%) |  |
| Age at first live birth | |  |  |  | 0.60 |
| <20 | | 654 (5.6%) | 637 (5.7%) | 17 (4.5%) |  |
| 20-24 | | 767 (6.6%) | 745 (6.6%) | 22 (5.8%) |  |
| 25-29 | | 2478 (21.4%) | 2387 (21.3%) | 91 (23.8%) |  |
| 30 and above | | 3397 (29.3%) | 3282 (29.3%) | 115 (30.1%) |  |
| Nulliparous | | 4167 (36.0%) | 4034 (36.0%) | 133 (34.8%) |  |
| Unknown | | 126 (1.1%) | 122 (1.1%) | 4 (1.0%) |  |
| Menopausal status | |  |  |  | 0.01 |
| Pre-menopausal | | 4493 (38.8%) | 4379 (39.1%) | 114 (29.8%) |  |
| Peri-menopausal | | 445 (3.8%) | 427 (3.8%) | 18 (4.7%) |  |
| Post-menopausal | | 6323 (54.6%) | 6085 (54.3%) | 238 (62.3%) |  |
| Unknown | | 328 (2.8%) | 316 (2.8%) | 12 (3.1%) |  |
| Menopausal hormone therapy use | |  |  |  | 0.01 |
| Never or Unknown | | 10215 (88.1%) | 9898 (88.3%) | 317 (83.0%) |  |
| Ever | | 1374 (11.9%) | 1309 (11.7%) | 65 (17.0%) |  |
| Personal history of benign breast disease | |  |  |  | 0.74 |
| No | | 1083 (9.3%) | 1032 (9.2%) | 51 (13.4%) |  |
| Yes | | 150 (1.3%) | 142 (1.3%) | 8 (2.1%) |  |
| Unknown | | 10356 (89.4%) | 10033 (89.5%) | 323 (84.6%) |  |
| Mammographic density | |  |  |  | 0.23 |
| Almost entirely fatty | | 1545 (13.3%) | 1506 (13.4%) | 39 (10.2%) |  |
| Scattered fibroglandular | | 5532 (47.7%) | 5350 (47.7%) | 182 (47.6%) |  |
| Heterogeneously dense | | 3446 (29.7%) | 3326 (29.7%) | 120 (31.4%) |  |
| Extremely dense | | 1066 (9.2%) | 1025 (9.1%) | 41 (10.7%) |  |
| Result of biopsy | |  |  |  | 0.01 |
| No prior biopsy | | 10143 (87.5%) | 9831 (87.7%) | 312 (81.7%) |  |
| Prior biopsy but diagnosis unknown | | 1296 (11.2%) | 1234 (11.0%) | 62 (16.2%) |  |
| Atypical hyperplasia | | 150 (1.3%) | 142 (1.3%) | 8 (2.1%) |  |
| First-degree relatives with breast cancer | |  |  |  | <.01 |
| 0/NA | | 9701 (83.7%) | 9410 (84.0%) | 291 (76.2%) |  |
| 1 | | 1760 (15.2%) | 1678 (15.0%) | 82 (21.5%) |  |
| 2 and above | | 128 (1.1%) | 119 (1.1%) | 9 (2.4%) |  |
| Second-degree relatives with breast cancer | |  |  |  | 0.90 |
| 0/NA | | 8813 (76.0%) | 8525 (76.1%) | 288 (75.4%) |  |
| 1 | | 2309 (19.9%) | 2232 (19.9%) | 77 (20.2%) |  |
| 2 and above | | 467 (4.0%) | 450 (4.0%) | 17 (4.5%) |  |
| Note—Unless otherwise indicated, data are number of patients with percentages in parentheses. Age is recorded in years.  ^*^ Data are means ± standard deviation, with range in parentheses.  ^†^ Breast cancer diagnosis included invasive disease only.  ^‡^ Comparing women with no breast cancer diagnosis to those with a breast cancer diagnosis within 5 years. | | | | | |

**Supplementary Figure 1**. **Flow chart of patient selection criteria.**

Used for model validation

Used for risk model comparison

^1^ 48,980 distinct patients verified from the total of 89,173 valid surveys.

^2^ Inclusion criteria for risk models: Women age in 40-74 years, without history of breast cancer, breast augmentation, or mastectomy.

^3^ Criteria for minimum data: the records met the criteria for minimum data if they contained the information on the following four risk factors: age, age at menarche, breast density, and whether there was a history of breast biopsy.

**Supplementary Figure 2. Calibration plots by decile of risk for 5-year predictions.**

| **BCRAT** | **BCSC** |
| --- | --- |
| 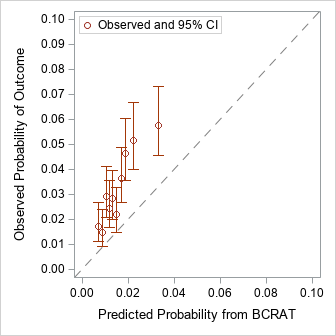 | 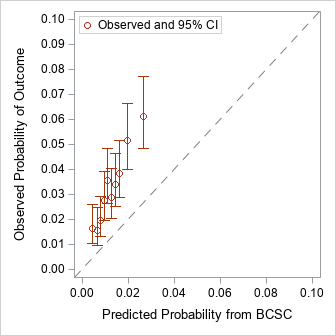 |
| **IBIS** |  |
| 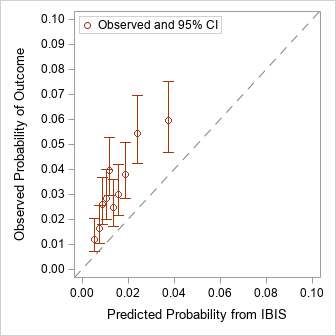 |  |
